# Light Steel-Timber Frame with Composite and Plaster Bracing Panels

**DOI:** 10.3390/ma8115386

**Published:** 2015-11-03

**Authors:** Roberto Scotta, Davide Trutalli, Laura Fiorin, Luca Pozza, Luca Marchi, Lorenzo De Stefani

**Affiliations:** Department of Civil, Environmental and Architectural Engineering, University of Padova, Via Marzolo 9, Padova 35131, Italy; roberto.scotta@dicea.unipd.it (R.S.); davide.trutalli@dicea.unipd.it (D.T.); laura.fiorin@dicea.unipd.it (L.F.); luca.marchi@dicea.unipd.it (L.M.); lorenzo.destefani@dicea.unipd.it (L.D.S.)

**Keywords:** steel-timber structures, light-frame structures, innovative technoprene bracing system, seismic design, behavior factor

## Abstract

The proposed light-frame structure comprises steel columns for vertical loads and an innovative bracing system to efficiently resist seismic actions. This seismic force resisting system consists of a light timber frame braced with an Oriented Strand Board (OSB) sheet and an external technoprene plaster-infilled slab. Steel brackets are used as foundation and floor connections. Experimental cyclic-loading tests were conduced to study the seismic response of two shear-wall specimens. A numerical model was calibrated on experimental results and the dynamic non-linear behavior of a case-study building was assessed. Numerical results were then used to estimate the proper behavior factor value, according to European seismic codes. Obtained results demonstrate that this innovative system is suitable for the use in seismic-prone areas thanks to the high ductility and dissipative capacity achieved by the bracing system. This favorable behavior is mainly due to the fasteners and materials used and to the correct application of the capacity design approach.

## 1. Introduction

Light timber-frame structures are widespread in the USA but this structural system is gaining acceptance worldwide because of the rapidity of realization, affordability, flexibility in design and construction, and good energy and structural performance. In seismic-prone regions such as Northern America, Italy, Japan and New Zealand, the application of this wall system as a Seismic Force Resisting System (SFRS) proved to be very efficient, thanks to its lightness and intrinsic dissipative capacity, if connections are correctly designed (e.g., [1]). In timber frame buildings, energy dissipation is mainly demanded to connections between bracing panels and timber frame, such as nails, screws or staples. Several works were conduced with the aim of analyzing the role of connections on the load-bearing capacity and deformation of timber-frame systems subjected to lateral loads. Recent works propose analytical models to predict their behavior [2,3,4]. Germano *et al.* [5] presented experimental results regarding the contribution of connections to the hysteretic behavior and energy dissipation capacity of the tested walls. Shake-table tests on full-scale light-frame buildings were also realized in Japan with the aim of evaluating dynamic properties of these timber constructions [6]. In [7] post-and-beam timber buildings braced with nailed shear walls are analyzed and different behavior factor values are proposed, depending on a building’s configuration and on the effect of different nail distributions at each storey. 

Innovative structural systems are commonly proposed in response to the changing needs of users and the construction industry, with the aim of optimizing the performance of traditional buildings [8]. For example, the coupling of timber with steel elements allows taking advantage of their intrinsic properties and to reduce their limitations, with the effect of improving the overall behavior of the building. Steel and wood can be integrated at component and/or building system level (e.g., steel connections with timber frames or walls, hybrid frames, steel frames and wood diaphragms) [9,10]. Examples of hybrid building systems were already realized and tested. Steel beams or frames combined with cross-laminated timber panels [11,12] or with timber-frame shear walls [13,14] have been studied through experimental tests and numerical modeling. These systems showed a relatively high ductility and demonstrated to be a reliable SFRS.

In light timber-frame systems, non-wood materials such as gypsum and cement plaster are also used as bracing components. The influence of these brittle materials on the performance of wood-frame shear walls is reported in [15].

Specially designed structural skins are commonly used as strengthening technique, e.g., reinforced-cement coating (jacket) for damaged masonry walls. In this case, a reinforced-cement layer is applied on the outer side or on both sides of the wall and it is connected to the masonry with steel anchors [16]. These exterior reinforcement techniques were also applied to light timber-frame systems. Zisi [17] and Zisi and Bennett [18] studied a system where the strengthening element is an anchored brick veneer tied to the exterior wall face of the wood-frame wall. A gypsum wallboard sheathing is added to the interior wall face. Analytical models demonstrated that both brick veneer and wallboard sheathings stiffen significantly the timber-frame shear wall. Results from shake-table tests [19] showed the influence of wall finish materials and gypsum interior wallboard on the behavior of light-frame wood constructions.

The use of novel systems in seismic areas require the assessment of mechanical properties through experimental tests in order to evaluate their seismic performance [20]. Van de Lindt [21] presented a summary of testing and modeling studies on timber shear walls over the last two decades of 20th century. More recently, in the United States, the seismic behavior of typical light-frame wood structural systems has been studied [22] to analyze the design and retrofit of existing wooden frame dwellings [23].

In this work, an innovative timber shear-wall system is presented. The light timber-frame system is coupled with an Oriented Strand Board (OSB) panel and an innovative technoprene slab infilled with plaster that improves not only the static and seismic behavior but also the insulation properties of the wall. The vertical load resistance is demanded to multi-storey thin-box steel columns that also allow to reduce the on-site construction time. They are fastened to the vertical panels, the foundations and the floors with steel brackets. Two walls were analyzed with quasi-static cyclic-loading tests according to EN 12512 protocol [24]. Then, numerical simulations allowed analyzing the behavior of a case-study building.

## 2. Description of the System

The proposed construction system combines a modular light timber frame with thin-box steel columns and an innovative external bracing system. The system represents an evolution of that described by Pozza *et al.* [25] where the outer reinforced concrete shelter is substituted by suitably shaped plastic panels infilled with plaster and the timber columns are replaced by steel ones.

**Figure 1:**
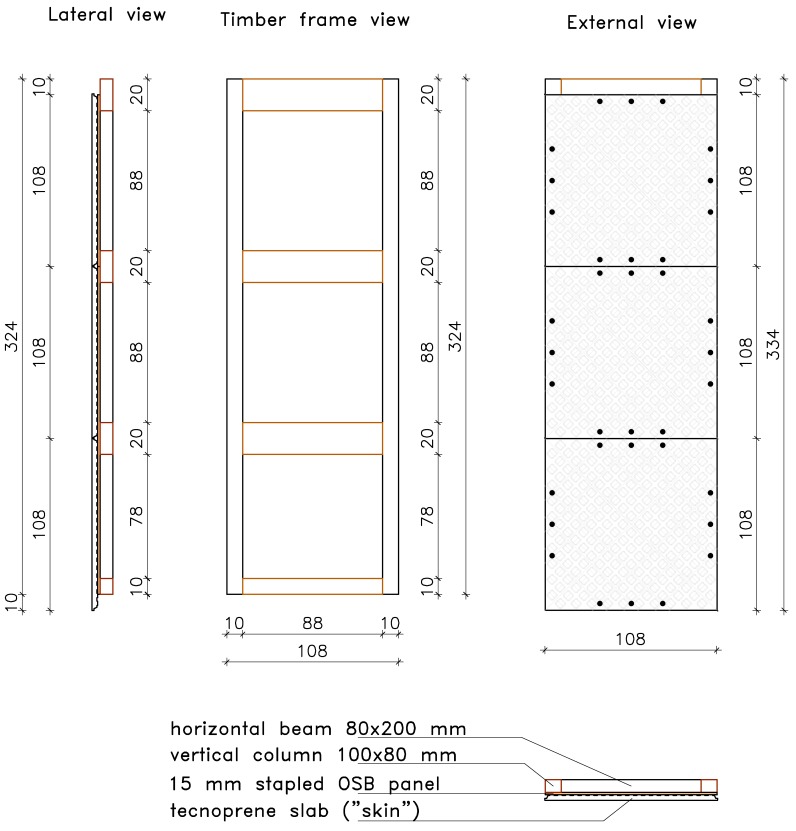
View of the modular shear wall (where not specified dimensions are in cm).

In this system, the structural elements have different functions: steel columns support live and dead vertical loads, whereas the OSB panel and the external plastic slab confer to the timber frame resistance against wind and earthquake actions. The frame (see Figure 1) has modular dimensions: width is equal to 1080 mm and height is three times the width dimension. Fifteen-millimeter thick OSB/3 panel conforming to EN 300 [26] is stapled to the timber frame, realized with 200 mm × 80 mm horizontal crosspiece beams and 100 mm × 80 mm vertical studs. Both beams and studs are made of C24 timber (EN 338) [27].

**Figure 2:**
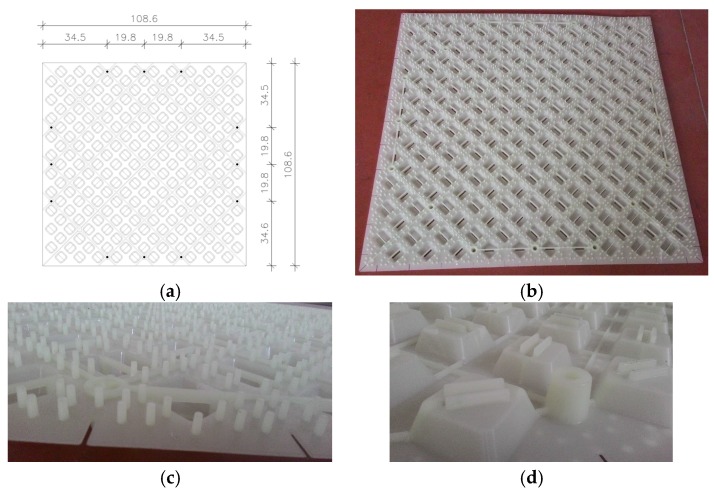
Technoprene slab (skin): (**a**) Geometry (cm) and fixing points; (**b**) Front view; (**c**) Front view in detail; and (**d**) Back view in detail.

The innovative technoprene slab (polypropylene homopolymer reinforced with 18.5% chemically coupled glass fiber), hereafter called skin (see Figure 2), is infilled with plaster and acts as an additional bracing system, collaborating with the OSB panel to provide strength and dissipative capacity to the timber frame. The skin is a square slab of about 108.6 mm width, and thickness equal to 35 mm, including the plaster layer. It is connected to the frame with three 10 mm × 120 mm screws on each side (steel class 8.8, according to ISO 898 [28]). 

The main advantages are:

1. The lightweight panel facilitates the realization of the buildings.
2. The special 3D shape improves the adherence with the plaster and allows the creation of a ventilation chamber between the OSB and the external layer (*i.e*., a continuous natural airflow from the ground level to the roof), improving the durability of the wooden parts and providing good insulation properties to the building.

**Figure 3:**
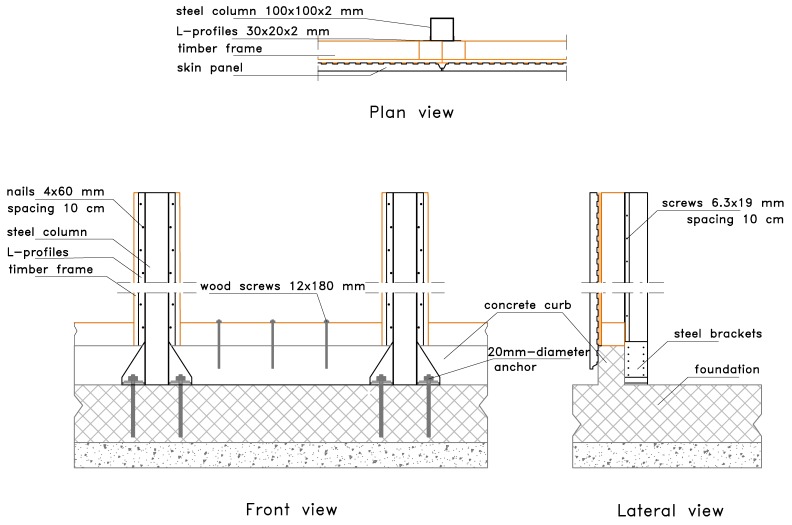

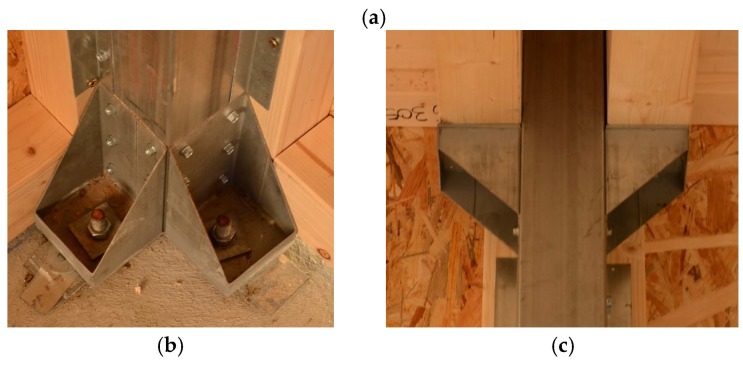
Steel column and brackets: (**a**) Geometric details of connection with foundation (dimensions in mm); (**b**) Connection with the foundation; and (**c**) Connection with the roof.

The steel columns (Figure 3) allow speeding up the construction process and assure resistance to vertical loads. In detail, the columns are placed before the shear walls and can be continuous from the foundation to the roof, for low- and medium-rise buildings. In this way, the assembly of the building is optimized in terms of rapidity and on-site management costs. Columns are connected to the frame with 30 mm × 20 mm × 2 mm press-belted L-profiles (continuous along the pillar), which are jointed to the wooden side with 4 mm × 60 mm ring shank nails and to the steel side with 6.3 mm × 19 mm screws (self-tapping screws according to EN 15480 [29], steel class 9.8 according to ISO 898 [28]). The same column-to-frame L-profiles have the function of connecting two adjacent modular shear walls. Therefore, two adjacent modules are indirectly jointed through a steel column.

**Figure 4:**
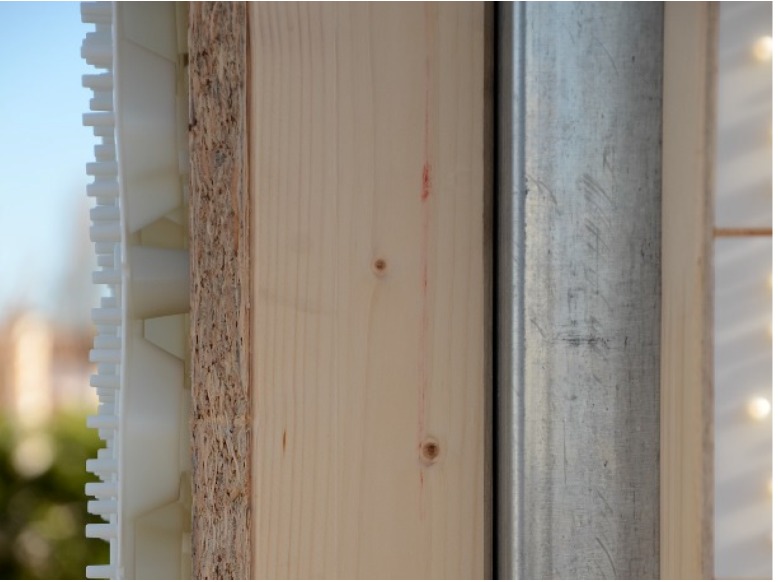
Wall section: (from left to right) skin, Oriented Strand Board (OSB) panel, timber stud, and steel column.

Connection elements, made with steel brackets, are used for supporting floor and roof beams and connecting columns at foundation, in order to resist to uplift of the shear wall. These brackets are made of the same tubular element of the column, 2- or 3-mm thick, and are connected with 6.3 mm × 19 mm self-tapping screws to the column and with 20 mm-diameter anchors to the foundation (see Figure 3). The resistance to base shear forces is provided mainly by three vertical 12 mm × 180 mm wood screws (class 4.8 according to DIN 571 [30]) fixed between the timber frame and the concrete foundation curb. Moreover, three horizontal 12 mm × 100 mm wood screws connect the bottom edge of the skin and the foundation curb. A vertical section of the system is shown in Figure 4.

## 3. Experimental Test

### 3.1. Test Setup and Procedure

Quasi-static cyclic-loading tests were conducted on two walls realized with the studied construction system, in order to assess the resistance of the system against lateral loads and to evaluate its seismic behavior.

The first wall tested (Wall A) was realized with all the components of the system. In the second wall (Wall B), the OSB panel was removed and only the skin acted as bracing system. In this way, the contribution of the skin to the global seismic response has been evaluated.

**Figure 5:**
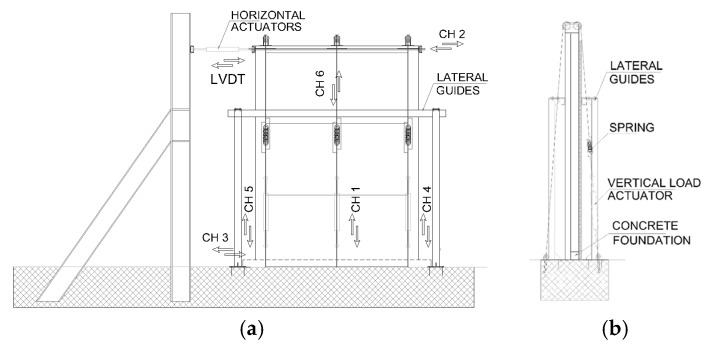
Test setup: (**a**) Frontal view and (**b**) Side view.

Two adjacent panels were assembled to realize the walls, which are 3.24 m high and 2.16 m wide. Tests were conducted subsequently with the same setup and instrumentation. A reinforced concrete foundation was realized to reproduce the base connection of the system. Figure 5 shows the test set-up used for both walls, which was chosen to be consistent with the previous experimental campaign, whose results are presented in [25]. A vertical load of 8.8 kN (reproducing gravitational loads at the first storey of a low-rise building with lightweight floors) was applied for each steel column by three hydraulic actuators. Lateral guides were positioned at the top of the specimen to avoid out-of-plane movement.

**Figure 6:**
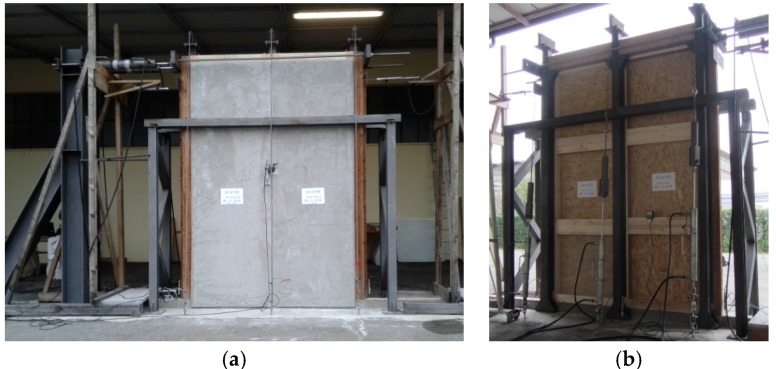
Experimental setup: external (**a**) and inner (**b**) views.

Displacements of panels and connections were measured with transducers, placed as shown in Figure 5: CH1, CH4 and CH5 measured the base uplift; CH3 the base slip; CH6 the panel-to-panel slip; CH2 the top displacement; and Linear Variable Displacement Transducer (LVDT) applied and measured the top horizontal force and displacement. Figure 6 shows the configuration before the test.

**Figure 7:**
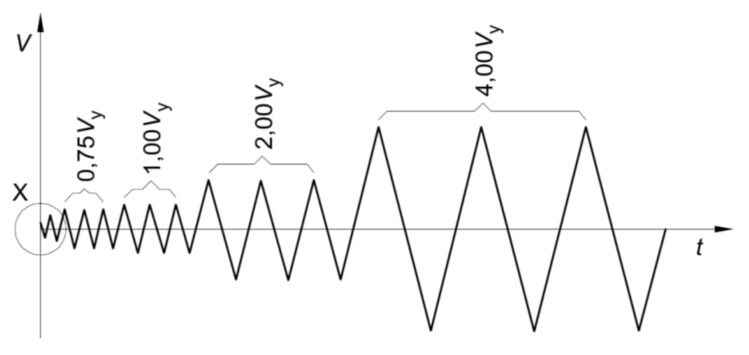
EN 12512 protocol [24].

Quasi-static cyclic-loading tests were performed in displacement control, according to EN 12512 [24], following the protocol shown in Figure 7. Such testing protocol requires the definition of the yielding displacement *V_y_* of the specimen assumed equal to 10 mm. Displacement was applied at a 0.2 mm/s rate. 

### 3.2. Test Results

**Figure 8:**
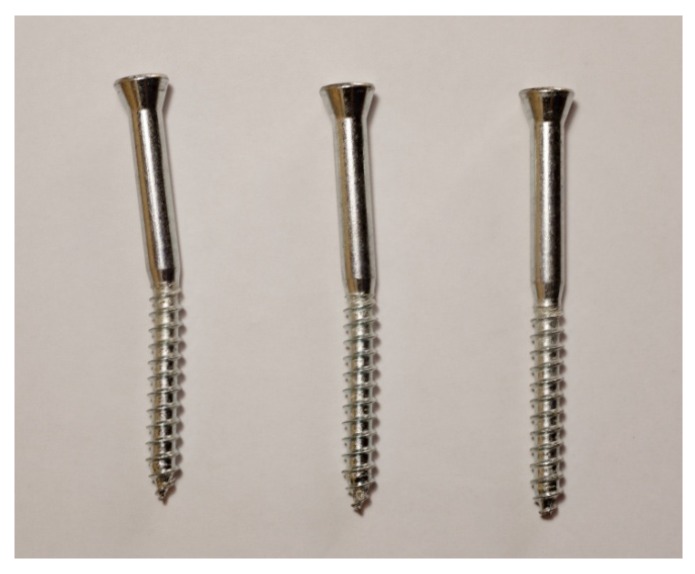
Yielding of the 10 mm × 120 mm screws connecting skin to frame (single plastic hinge).

**Figure 9:**
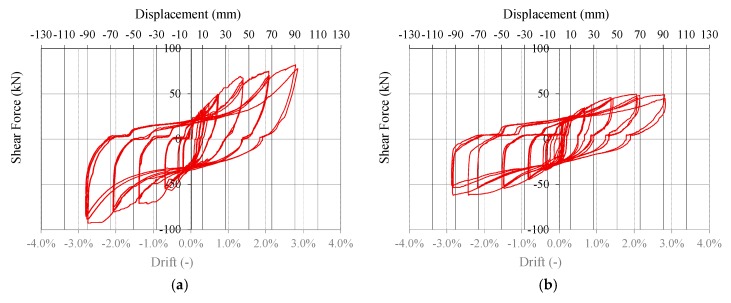
Hysteresis curves: (**a**) Wall A and (**b**) Wall B.

**Figure 10:**
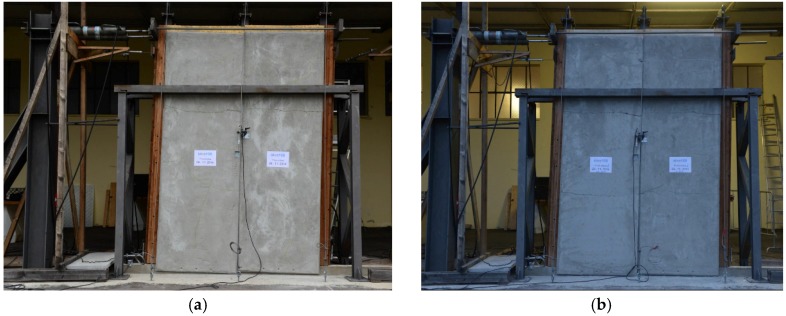
Wall configurations at the end of the cyclic loading tests: (**a**) Wall A and (**b**) Wall B.

At the end of the tests, no failure localization was evident, but there was diffuse yielding of fasteners between bracing systems and frame and between frame and steel *L*-profiles. Thin cracks at the perimeters of the skin panels were also observed. Figure 8 demonstrates the formation of a plastic hinge in the 10 mm × 120 mm wood screws connecting skin to frame. Tests were stopped before the ultimate displacement of the walls was reached, due to limited allowable jacket elongation. Figure 9 shows the hysteresis curves of the two specimens, *i.e.* the imposed top displacement *vs.* the corresponding applied force. Figure 10 shows the walls at the end of the tests, at the maximum applied displacement. 

Wall A reached the maximum displacement without relevant failures or strength degradation phenomena (Figure 9a). This specimen exhibited the hysteretic behavior typical of timber structures, characterized by pinching phenomenon of steel–wood and wood–wood connections. Moreover, the skin and the OSB panel contributed to the hardening behavior shown.

The shear resistance of the system is limited by the weakest mechanism among the followings:

1. The in-plane shear resistance of skin and OSB panel and the shear resistance of the relative connectors.
2. The axial and shear resistance of the connections at foundation.
3. The shear resistance of frame-to-column joints.

The main contributions to the ductility of the system are given by the shear deformation of the bracing system and the panel-to-panel relative slip (see Figure 10a). Conversely, base connections should be over-designed due to their brittle behavior and, therefore, small and almost elastic deformations are expected for them, according to the capacity design approach. 

Figure 9b allows assessing the contribution of the skin to the shear resistance of the whole system. In the cyclic tests for Wall A and Wall B, the same displacements were reached with lower resistance for Wall B. The hysteretic behavior of this wall also confirms the contribution of the skin panel to the energy dissipation capability of the system: the pinching behavior was reduced and the ductility was maintained. The comparison between Figure 9a,b also allows quantifying this contribution in terms of strength: it can be stated that almost the 60% of in-plane shear resistance is given by the skin.

### 3.3. Analysis of Experimental Results

Results obtained from the cyclic tests were analyzed to define the main mechanical properties of this innovative system. This section discusses the evaluation of the following parameters: yielding point (*V_y_*, *F_y_*), maximum displacement and force reached (*V*_max_, *F*_max_), stiffness for the elastic and post-elastic branches (*k*_e_, *k*_p_), ductility *μ*, strength degradation at different cycle amplitudes, and viscous damping ratio *ν*_eq_. Finally, the evaluation of the ductility class, according to design provisions [31] is reported.

#### 3.3.1. Ductility Estimation

Table 1 and Table 2 show the evaluation of yielding points and the main outcomes in terms of strength, stiffness and ductility according to different approaches. These parameters are defined using suitable bi-linearization methods of the envelope force–displacement curve, which is generally not regular and does not exhibit well-defined yielding condition. 

The fact is that various methods are proposed to compute this point [24,32]. The first method proposed by EN 12512 [24] is adequate for curves with two well-defined linear branches. The yielding point is defined by the intersection of these two lines. The second method proposed by EN 12512 [24] assumes the hardening stiffness equal to 1/6 of the elastic stiffness, without taking into account the actual hardening slope. Alternative methods to determine the yielding point are based on an energy approach. The equivalent Elastic–Plastic Energy method (EEEP) [33] is based on balancing the strain energy between the actual curve and the bi-linear one, and is characterized by a perfectly-plastic post-elastic branch. Another one is an equivalent-energy method with post-elastic hardening branch and is indicated as EEEH [20].

**Table 1:** Wall A test results and interpretation according to EN 12512 method (ENa) [24], equivalent Elastic–Plastic Energy method (EEEP) [33] and Equivalent-Energy Method with Post-Elastic hardening branch (EEEH) [20].

Parameters	Notations (units)	ENa	EEEP	EEEH
Ultimate displacement	*V*_max_ (mm)	92.0	92.0	92.0
Ultimate force	*F*_max_ (kN)	86.0	65.9	86.0
Elastic stiffness	*k*_e_ (kN/mm)	7.5	4.5	5.0
Hardening stiffness	*k*_p_ (kN/mm)	0.5	0.0	0.5
Yielding displacement	*V_y_* (mm)	5.6	14.7	8.6
Yielding force	*F_y_* (kN)	41.7	65.9	43.2
Ductility ratio	*μ* = *V_u_*/*V_y_* (-)	16.5	6.2	10.7

**Table 2:** Wall B test results and interpretation according to equivalent Elastic–Plastic Energy method (EEEP) [33].

Parameters	Notations (units)	EEEP
Ultimate displacement	*V*_max_ (mm)	90.0
Ultimate force	*F*_max_ (kN)	47.4
Elastic stiffness	*k*_e_ (kN/mm)	4.1
Hardening stiffness	*k*_p_ (kN/mm)	0.0
Yielding displacement	*V_y_* (mm)	11.6
Yielding force	*F_y_* (kN)	47.4
Ductility ratio	*μ* = *V_u_*/*V_y_* (-)	7.7

In this work, the envelope of the hysteresis curve was fitted using the analytical formulation proposed by Foschi and Bonac [34]. Then, the mechanical parameters were obtained applying the bi-linearization methods that better fit the envelope curve. In detail, results for Wall A were fitted with ENa, EEEP and EEEH methods. To obtain a comparison with Wall B in terms of stiffness and strength, only the EEEP method was applied due to its perfectly-plastic behavior. In fact, EN and EEEH methods cannot adequately fit elastic–perfectly plastic envelope curves and therefore they may provide inconsistent results when treating with curves without well-defined hardening behavior.

The use of different approaches causes a variation of both yielding displacement and force, due to the variation of the elastic and post-elastic stiffness. This means that the ductility value could be strongly influenced by the method of bi-linearization used. In particular, the EEEP method normally over-estimates the yielding force [32], whereas EN methods are generally less conservative in terms of ductility than energy-based ones.

Ductility ratios were evaluated assuming the maximum applied displacement as ultimate top displacement, *i.e.*, 92 mm for wall A and 90 mm for Wall B.

Obtained ductility is always higher than 6, which is the minimum value to be assured for the High Ductility Class (HDC), according to Eurocode 8 [31]. Comparing the obtained ductility values with other studies [35,36], it can be seen that the ductility of this novel system is greater than that obtained for massive timber systems (e.g., Cross-laminated Timber—CLT). This is because the shear deformation of the bracing panels allows the achievement of higher ductility than shear walls, in which the displacement capacity is mainly concentrated in the base connections. 

#### 3.3.2. Strength Degradation and Viscous Damping Ratio

Timber structures assembled using metal fasteners are sensitive to stiffness and strength degradation of connection elements when undergoing cyclic action. The consequent strength reduction is an important parameter to identify the ability of a structure to resist cyclic action and therefore seismic shocks. According to Eurocode 8 [31], this parameter and the ductility ratio are used to define the Ductility Class of a timber structure. Table 3 lists the strength degradation recorded between the first and third cycles of each displacement level of the tested walls and the equivalent viscous damping *ν*_eq_. These values were defined according to EN 12512 [24].

**Table 3:** Strength degradations and equivalent viscous damping at each cycle amplitude.

Cycle Amplitude (mm)	Strength Reduction (%)	*ν*_eq_ (%)
Wall A		
20	5.9	23.8
40	5.9	20.9
60	7.6	19.6
80	11.4	18.6
Wall B		
20	3.4	31.2
40	4.9	24.3
60	8.9	24.9
80	16.2	25.8

Values listed in Table 3 demonstrate that the loss in strength increases with the cycle amplitude. This value is always less than 20%; therefore, given the ductility higher than 6, the system can be classified as High Ductility Class (HDC).

Table 3 also lists the equivalent viscous damping *ν_eq_* values, which summarize the hysteretic dissipative capacity of a structural system. These values are constantly greater than 18%, confirming the good dissipative capability of this system. Moreover, it can be seen that the values of the equivalent viscous damping for Wall B are higher than results for the entire system (Wall A). These values confirm the contribution of the skin to the dissipative capability of the system and therefore its suitability for use in seismic areas. 

Comparing the results with those obtained for the previous system [25], a slight improvement in terms of both strength reduction and equivalent viscous damping has been obtained.

## 4. Numerical Simulations

In this section, the description of the numerical model adopted to simulate the seismic behavior of a case-study building and main results are reported and discussed. 

Reliable models of timber structures should take into account the mechanical properties of connection elements, which affect the global behavior of the building [37]. In detail, values of strength and stiffness of connections—derived from codes or tests—are sufficient for linear modeling, whereas post-elastic and hysteretic behavior of connections also has to be correctly considered to perform nonlinear dynamic simulations (NLDA). This type of analysis allows evaluating the intrinsic dissipative capacity and the intrinsic over-strength of the studied system and to obtain an estimation of the behavior factor—henceforth called “*q*-factor”—which summarizes such capabilities [38].

The main features of the model (geometry, type of elements and hysteretic behavior) are reported and the calibration based on experimental test data of Wall A is described. Finally, the analysis of a case-study building was conduced in order to obtain an estimation of the *q*-factor and to verify that the system belongs to HDC [31].

### 4.1. Nonlinear Model

The modeling approach for novel timber systems consists of the following steps [39]:

1. Execution of quasi-static cyclic loading tests, according to EN12512 [24], of an entire wall specimen representative of the studied system and recording of applied lateral force *vs.* displacement of wall and connections, as shown above.
2. Calibration based on test results of each nonlinear hysteretic spring representing connection elements and bracing system, in terms of equivalence of hysteresis cycles and dissipated energy for each element.
3. Assembling of linear and nonlinear elements to reproduce the tested wall specimen and to simulate the cyclic-loading test.
4. Comparison of numerical and experimental curves of the wall in terms of hysteresis cycles and energy dissipation, in order to validate the model.
5. Modeling of the case-study building and execution of NLDA.

**Figure 11:**
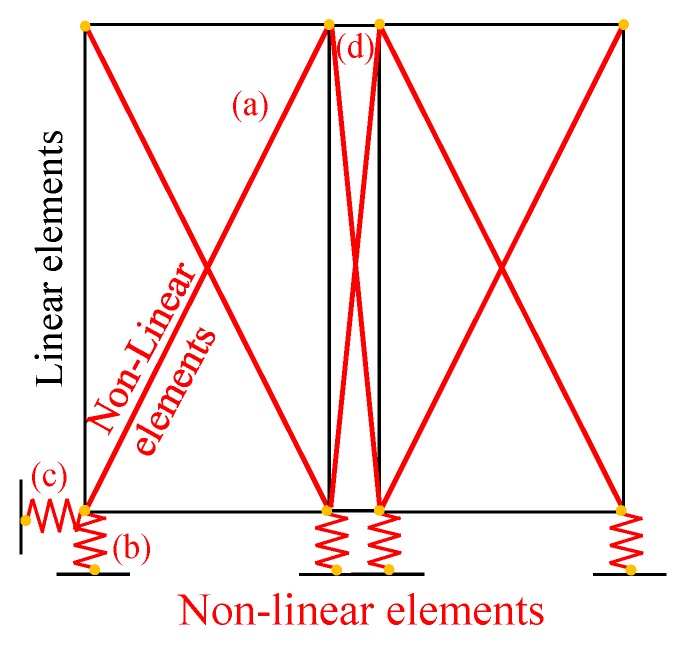
Finite Element model of Wall A for model validation.

**Figure 12:**
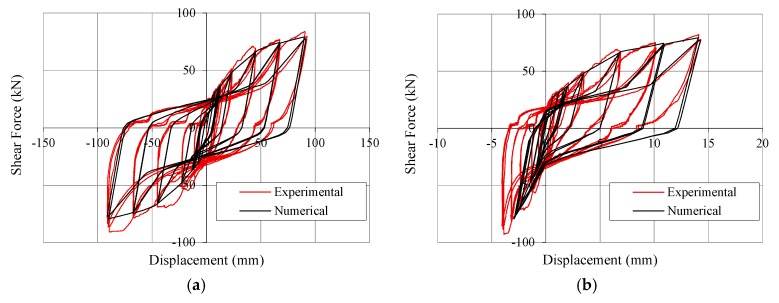

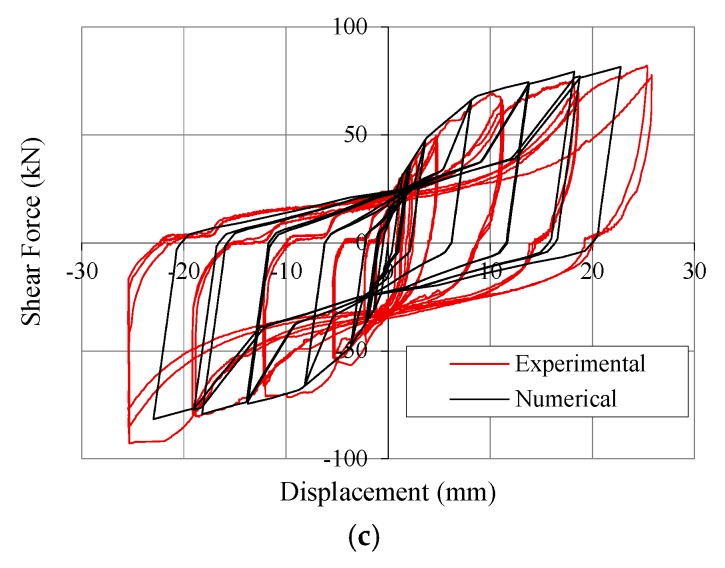
Hysteresis cycles of shear wall: (**a**) Top displacement *vs.* lateral force; (**b**) Displacement at hold-down *vs.* lateral force; and (**c**) Relative displacement at vertical joint *vs.* lateral force.

In the studied shear walls, both connection elements and bracing system are characterized by an hysteretic behavior and show pinched load-displacement responses and strength degradation under cyclic loading, which are typical for timber systems [37]. In order to reproduce faithfully their actual response, the research-oriented numerical code “Open System for Earthquake Engineering Simulation OPENSEES” [40] was used. The hysteresis material model Pinching4 proposed by Lowes and Altoontash [41] was adopted for each non-linear elements. This model allows replicating the monotonic nonlinear curve with four slopes, the pinching behavior and strength and stiffness degradation phenomena. It requires the calibration of sixteen parameters for stress and strain on the positive and negative response envelopes; six parameters for pinching cycles; and four parameters for strength degradation. Stiffness degradation was not considered. The main modeling hypotheses are that nonlinear behavior is concentrated in the connection and bracing systems, whereas the wood frame remains elastic. Figure 11 shows the Finite Element (FE) model of the tested shear wall. Each finite-element module consists of a perimeter frame made with elastic trusses braced by diagonal nonlinear springs (Figure 11—element a), which reproduce faithfully the in-plane cumulated response of stapled OSB panel, plastic skin and relative connectors (staples and screws). Inelastic springs are also used for hold-downs (element b), base shear bolts (element c) and in-plane vertical joints between adjacent wall modules (element d). Table 4 lists the main parameters for each nonlinear element. Linear compression-only elements are coupled in parallel with hold-down springs in order to simulate the asymmetric behavior of this component, as shown in Figure 12. Vertical loads and seismic masses are applied at upper nodes. For further details on this modeling strategy, see [25].

**Table 4:** Main parameters of non-linear springs representing the connections.

Main Parameters	Units	Bracing Panel (a)	Hold-Downs (b)	Base Shear Screws (c)	Vertical Joints (d)
Elastic stiffness	kN/mm	7.00	64.04	617.50	33.25
First yielding force	kN	14.00	38.42	24.70	26.60
Post-elastic stiffness	kN/mm	1.22	11.70	105.55	6.48

### 4.2. Simulation of Tests

**Figure 13:**
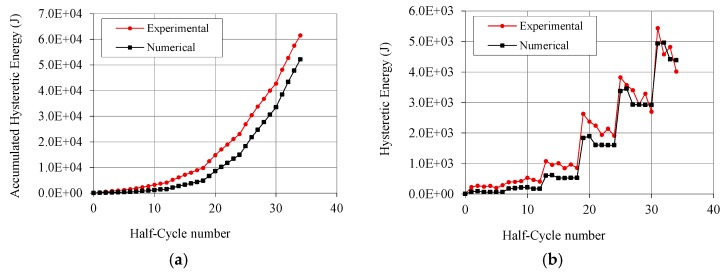
Energy comparison: (**a**) Accumulated hysteresis energy up to the end of the test; and (**b**) Dissipated energy computed for each half-cycle.

After the calibration of the elementary nonlinear connections, the experimental cyclic test of Wall A described above was reproduced with the numerical model in Figure 11, by imposing the same horizontal top displacements (loading protocol according to EN 12512 [24]) and vertical load and recording displacements at the same position of test transducers (Figure 5). The good accuracy of the model was ascertained by comparing the numerical data with test results. Figure 12 shows main recorded results superimposed on experimental cycles, *i.e.*, lateral force *vs.* displacement at the top (Figure 12a), *vs.* displacement at hold-downs (Figure 12b) and *vs.* relative displacement at vertical joint (Figure 12c). The dissipated energy graphs (Figure 13) clearly show that the numerical model never over-estimates the experimental values, and that the difference between numerical model and test data in the near-collapse condition is less than 10% (Figure 13a). Figure 13b also shows the dissipated energy computed separately for each pulling and pushing phase (*i.e.*, half-cycle). These comparisons allow to validate the model in terms of strength, stiffness and hysteresis behavior of the shear wall (Figure 12a) and of hold-downs and vertical joints (Figure 12b,c). Moreover, the energy comparison in Figure 13 allows affirming that the estimated values of *q*-factor are reliable and conservative.

### 4.3. Case-Study Building and Design Criteria

**Figure 14:**
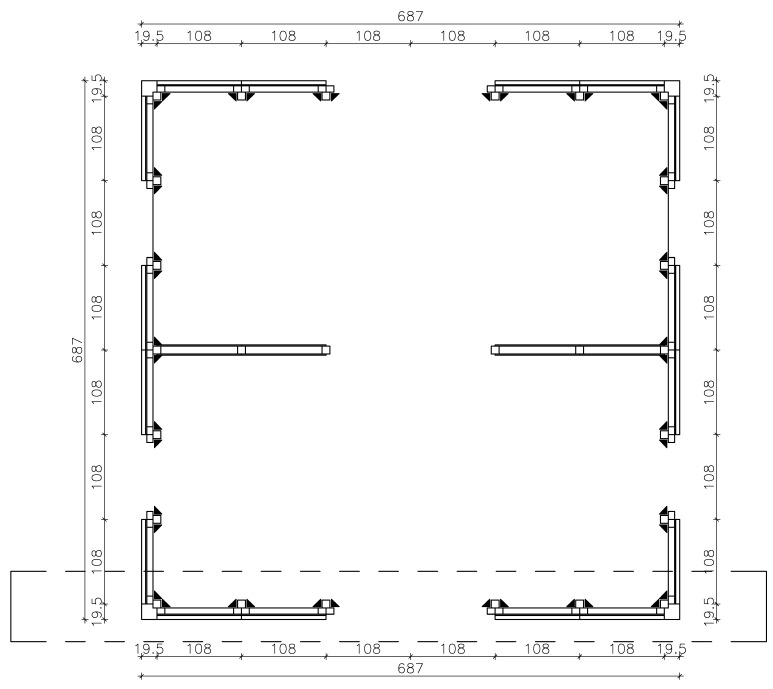
Case-study building: First-storey plan (dimensions in cm).

The three-storey CLT building tested on the shaking table during the SOFIE project [37,42] was assumed as the case study. A 2D model of the façade placed in direction *X* was analyzed (evidenced within dashed box in Figure 14). To allow a simplified 2D model of the structure, configuration with symmetric openings was chosen and rigid diaphragm assumption was made.

The same precast modular panels subjected to the cyclic test and numerical calibration described above were used in the model to assemble the building, conforming the resistance of the base connections to the seismic loads.

In order to evaluate the peak ground acceleration (PGA) compatible with an elastic design of the case-study building (PGA_d_) and to compare it with the PGA that leads the non-linear model to the near-collapse condition (PGA_u_), the elastic response spectrum for building foundations resting on type A soil (rock soil, corresponding to *S* = 1.0, *T*_B_ = 0.15 s, *T*_C_ = 0.4 s, *T*_D_ = 2.0 s), behavior factor *q* = 1, and building importance factor γ_I_ = 1 was assumed according to Eurocode 8 [31]. The maximum spectral amplification factor *F*_0_ was assumed equal to 2.5. Then, the unit lateral load-bearing capacity of the shear wall was deduced from the experimental load-displacement curve, *i.e.*, the force corresponding to the yielding of the shear wall (according to the EEEP bilinear model) was assumed to be the conventional design strength of the wall. Therefore, given the overall seismic mass equal to 25.2 t, the PGA_d_ compatible with an elastic design of the structure, without safety factors applied, was equal to 0.21 g, assuming the fundamental period of the shear wall within the plateau range. The hypothesis that the first mode period was in the plateau range was confirmed by the frequency analysis, which provided the fundamental period of the building *T*_1_ = 0.36 s.

### 4.4. Evaluation of q-Factor

The method proposed by Ceccotti and Sandhaas [39] was used to estimate the *q*-factor, obtained as ratio between PGA_u_ and PGA_d_. The applicability of such method requires some additional clarifications about the definition of the design and of the near-collapse condition. Differences between design and modeling phase introduce uncertainties in the final values of *q*-factor, which can be influenced mainly by design over-strength. A correct evaluation of the intrinsic *q*-factor should take into account all intrinsic capacities of the system, *i.e.*, dissipative capacity, ductility, redundancy, post-elastic hardening behavior, and strength reserve. However, each over-resistance of walls induced by design criterion (e.g., safety level assumed by designers or simplified analytical methods for design) should not influence this value and can be computed in addition.

In this work, the yielding condition was assumed coincident with design condition (*i.e*., PGA_d_ = PGA_y_), in order to evaluate the intrinsic value of the *q*-factor. It is clear that such design condition depends only on the bi-linearization method adopted to evaluate the yielding limit from the experimental load–displacement curve, whereas it is independent of the design of the structure. The actual over-design subfactor, *i.e.*, the ratio between PGA_y_ and PGA_d_, which can be obtained via codes and analytical methods, should be multiplied by the intrinsic *q*-factor to obtain actual overall *q*-factor of the building.

The near-collapse condition must be defined to compute the PGA_u_ with NLDA, assuming a criterion based on the maximum displacement capacity that the structure can reach without collapsing. Near-collapse limits were fixed as: (a) vertical uplift 18 mm and (b) inter-storey drift of bracing system of 2.0%.

A capacity design approach was followed in order to avoid brittle failures and to obtain the maximum ductility of the building at the near-collapse condition. Consequently, the weakest components of the structure were the bracing system and the vertical joint, *i.e.*, OSB panel-to-frame connections, skin-to-frame connections and frame-to-column connections, which in each test and analysis yielded before other seismic-resisting components, *i.e.*, before yielding of other connections and failure of wooden and plastic components. Therefore, obtained values of *q*-factor are consistent only with a correct capacity design of the building. Otherwise, the building could fail before reaching the maximum ductility and the PGA_u_ could be lower.

The NLDA were carried out considering eight seismic shocks, artificially generated with SIMQKE_GR [43] in order to meet the spectrum compatibility requirement with the design elastic spectrum. Dynamic equilibrium equations were integrated with a not-dissipative Newton–Raphson scheme and time-steps of 0.001 s, introducing an equivalent Rayleigh viscous damping of 2%, according to [37]. By progressively increasing the magnitude of the applied seismic signals, the PGA_u_ values, which lead to the near-collapse condition, were evaluated for all signals. Lastly, the *q*-factors for each signal were evaluated as the ratio between the PGA_u_ values and the PGA_d_ value. Results are reported in Table 5 and Figure 15, with average and 5% characteristic values (q_0.05_) computed according to EN 1990 [44]. The obtained average *q*-factor was 5.42, confirming the good dissipative capability of the tested system. The 5% characteristic value, equal to 4.62, could be used as conservative estimation of the intrinsic *q*-factor.

In recent works concerning light timber-frame buildings, values of *q*-factor equal to 2.5 [5] or in the range between 2.5 and 4.5 [7] were obtained. The innovative system here investigated assures higher values of *q*-factor due to the presence of staples and additional fasteners (skin-to-frame screws and frame-to-column nails), which diffusely yielded, providing high ductility and dissipation capacity to the system. 

**Table 5:** Obtained *q*-factor values.

Seismic Signals							
#1	#2	#3	#4	#5	#6	#7	#8
5.2	5.5	5.5	5.2	6.2	5.0	5.7	5.0

#: Earthquake.

**Figure 15:**
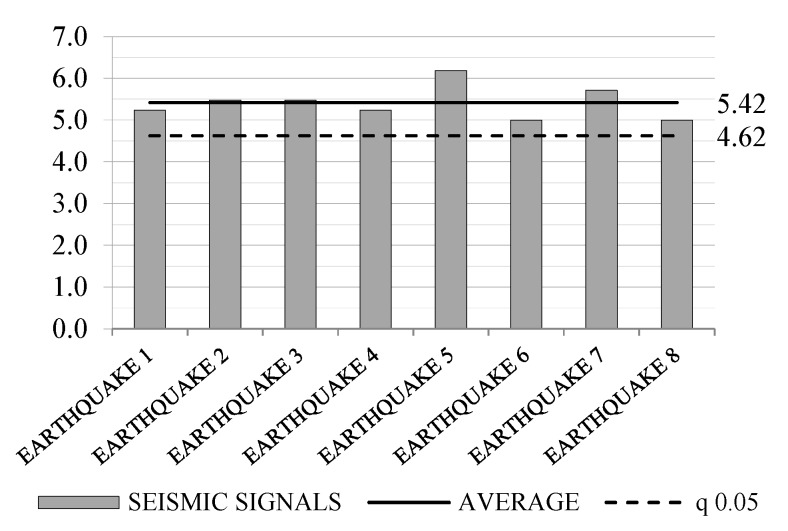
Obtained *q*-factor values, average value and 5% characteristic value.

## 5. Conclusions

Results from experimental tests and numerical simulations demonstrated that the proposed innovative construction system represents a viable technique for high-ductility construction in seismic-prone areas. 

Experimental results show that this steel-timber shear-wall system is characterized by a pronounced dissipative behavior if subjected to horizontal cyclic loads, thanks to the response of the bracing system, which is able to deform plastically for at least three fully reversed cycles, with high value of static ductility and limited reduction of resistance (less than 20%). These properties make this system classifiable in HDC.

Numerical results confirmed test evidence and the ductility class hypothesized. To design this system with linear analyses, a behavior factor up to 4.5 can be adopted if a rigorous capacity design approach is applied. In detail, all base connections and all brittle components (timber and plastic) must be over-resistant compared to the bracing system and the nails at vertical joints, which are the most ductile and dissipative components of the shear wall. A damage-limitation-state verification should also be conduced in order to limit the deformation of the system and to avoid unacceptable damage to the building.

Such results are based on the analysis of a single three-storey building. In order to generalize such results (e.g., variability of the *q*-factor with building characteristics), variations of the case-study building will be considered in future works. In addition, obtained results depend on applied design method, based on test data and on capacity design approach. A comparison with results obtained varying the adopted design method could lead to variations in the *q*-factor.
